# Pan-cancer analysis and mechanistic insights of tuft cell-like tumors

**DOI:** 10.1016/j.gendis.2025.101939

**Published:** 2025-11-14

**Authors:** Mengling Ye, Yuyang Liu, Hui Li

**Affiliations:** a Department of Pathology, School of Medicine, University of Virginia, Charlottesville, VA 22908, USA; b Department of Research, Guangxi Medical University Cancer Hospital, Nanning, Guangxi 530000, China

**Keywords:** Biomarkers, Cancer classification, POU2F3, Tuft cell, Tuft cell-like tumors

## Abstract

Tuft cells are rare, chemosensory epithelial cells present in various tissues, including the respiratory and gastrointestinal tracts. Recent studies have revealed their significant role in cancer biology, particularly through the expression of the transcription factor POU2F3, which serves as a master regulator of tuft cell lineage. In several cancer types, including small cell lung cancer, gastric cancer, and breast cancer, POU2F3 expression defines a distinct molecular subtype termed “tuft cell-like” tumors. These tumors exhibit unique transcriptional programs and altered tumor-immune interactions, contributing to their distinct therapeutic sensitivities. In this review, we first analyze the expression patterns of POU2F3 across cancer types using the TCGA datasets, revealing differential expression profiles and supporting the classification of tuft cell-like subtypes. We further explore cancer-type-specific signaling pathways regulating tuft cell differentiation and function, such as IL-25, acetylcholine, and taste receptor-related pathways. Finally, we propose that tuft cell-like signatures may serve as promising biomarkers for diagnosis, prognosis, and treatment stratification. Understanding the tuft cell-like–POU2F3 axis could open new avenues for targeted therapies in lineage-defined cancers.

## Introduction

Tuft cells are specialized chemosensory epithelial cells recognized for their distinct morphology, which includes long, thick microvilli on the apical surface.^1^ Initially identified in the trachea and gastrointestinal tract of rodents, tuft cells have since been found in a variety of organs, including the thymus, urethra, stomach, intestines, biliary tract, and respiratory airways, as well as more recently in pancreatic precancerous lesions.^2^^,^^3^ Tuft cells are uniquely equipped to sense a wide range of environmental chemical signals, which allows them to act as sentinels in various tissues. These cells are often located in areas exposed to external stimuli, such as the gastrointestinal tract, airways, and mucosal surfaces, where they can detect changes in the environment that may indicate infection, injury, or other pathophysiological events.^3^^,^^4^ Functionally, tuft cells play a pivotal role in orchestrating type 2 immune responses. Upon sensing environmental signals, they secrete interleukin-25 (IL-25), which activates group 2 innate lymphoid cells and promotes downstream immune responses critical for defense against helminth infections and modulation of mucosal inflammation.^5^, ^6^, ^7^ Emerging evidence has linked tuft cells to cancer biology, as multiple tumor types, including lung cancer, gastric cancer, pancreatic tumor, colorectal cancer, prostate cancer, and breast cancer, exhibit a tuft cell-like transcriptional signature characterized by the expression of canonical markers, such as POU class 2 homeobox 3 (POU2F3, a lineage-defining transcription factor essential for tuft cell specification), doublecortin-like kinase 1 (DCLK1), transient receptor potential cation channel, subfamily M, member 5 (TRPM5), Advillin (AVIL), choline O-Acetyltransferase (CHAT), Achaete-Scute family BHLH transcription factor 2 (ASCL2), cytochrome C oxidase subunit I (COX1), and SRY-box transcription factor 9 (SOX9).^8^, ^9^, ^10^, ^11^ Despite increasing recognition of tuft cell-like features in tumors, the exact role of tuft cells in tumorigenesis remains incompletely understood. Moreover, there is currently no standardized framework for classifying tuft cell-like tumors across different tissue types, underscoring the need for further investigation into their oncogenic potential and diagnostic relevance. Therefore, in this review, we first performed a preliminary classification of tumors based on POU2F3 expression using data from The Cancer Genome Atlas (TCGA) database to identify tumor types potentially associated with tuft cells. We then conducted a comprehensive literature review to summarize the reported functions and mechanistic roles of tuft cells across various tumor types, aiming to provide a reference for future research.

## Normal function of POU2F3

POU2F3 is a lineage-defining transcription factor essential for the specification of tuft cells and other TRPM5^+^ chemosensory epithelial cells.^12^^,^^13^ Functional studies using *Pou2f3* knockout mice have shown that the loss of POU2F3 results in a marked deficiency of intestinal tuft cells, leading to impaired type 2 immune responses, reduced goblet cell hyperplasia, and delayed clearance of parasitic infections.^5^^,^^14^ In the gastrointestinal tract, POU2F3-driven tuft cells play a key role in orchestrating immune responses and epithelial remodeling during helminth infection. *Pou2f3*-deficient mice exhibit delayed expulsion of *Hymenolepis diminuta*, reduced goblet cell responses, and slower intestinal transit, although systemic Th2 responses remain largely unaffected.^14^ In the thymus, POU2F3 is highly expressed in thymic tuft cells and Late Aire 2 precursors, where it regulates the expression of tuft cell-specific genes associated with increased chromatin accessibility, highlighting an epigenetic mechanism of lineage specification.^15^ Beyond the immune and gastrointestinal systems, POU2F3 also plays a critical role in the development and maintenance of taste receptor cells, including sweet, umami, and bitter taste receptor cells in the oral epithelium,^16^ further emphasizing its conserved role in chemosensory lineages.

## POU2F3-based classification of tuft cell-like tumors

Although research on tuft cell-associated tumors has advanced significantly in recent years, a standardized pan-cancer classification based on tuft cell-like gene expression signatures remains underdeveloped. A previous study analyzed TCGA datasets and immunohistochemistry data to explore the presence of tuft cell-like tumors across extra-thoracic cancer types.^17^ While this work offered valuable insights, it was limited by the lack of sufficient functional characterization and incomplete integration with findings from other tumor types. To address these limitations, we propose a more comprehensive classification strategy that combines transcriptomic data from TCGA with functional and mechanistic evidence from existing literature. This integrated approach aims to improve the understanding and therapeutic targeting of tuft cell-associated tumors across cancer types.

In this review, we focus on POU2F3, a master regulator of tuft cell differentiation, as the core marker for identifying tuft cell-like tumors. POU2F3 is essential for tuft cell lineage specification and exhibits strong tumor-type specificity, making it a reliable and consistent biomarker.^18^ While other tuft cell-associated genes, such as growth factor independent 1B (GFI1B), TRPM5, SOX9, CHAT, and AVIL, are also linked to tuft cell identity, their expression is often context-dependent and less stable across tumor types. Relying on POU2F3 alone reduces variability and improves classification clarity, avoiding the complexity introduced by multi-marker panels. Additionally, we examine the biological significance of tuft cell-like gene expression in cancer, particularly its roles in immune regulation and tumor progression. We also discuss its potential as a diagnostic marker and therapeutic target, highlighting tuft cells as an emerging player in tumor biology. Gene expression and survival analyses were conducted using gene expression profiling interactive analysis (GEPIA; http://gepia.cancer-pku.cn), which integrates TCGA datasets for standardized comparison.

## Classification based on TCGA data analysis

In this study, we systematically analyzed POU2F3 expression patterns across TCGA pan-cancer data and found that POU2F3 was broadly expressed in most tumor types, with log_2_(TPM+1) > 0 in nearly all cancer tissues. To further explore expression differences, we classified tumors based on the comparison between cancer and corresponding normal tissues into three groups: i) tumors with higher POU2F3 expression in cancer tissue than in normal tissue, ii) tumors with lower expression in cancer tissue, and iii) tumors with no significant difference between the two. Subsequently, we reviewed existing literature to understand the potential mechanisms of tuft cell involvement across different tumor types, focusing on their context-dependent roles in promoting or suppressing tumorigenesis. For example, in some cancers, tuft cells may facilitate cancer progression by modulating the immune microenvironment or influencing cancer cell metabolism, while in others, tuft cells may suppress tumorigenesis through the secretion of specific molecules. This classification enhances our understanding of tuft cell function in cancer and informs potential therapeutic targeting strategies. Additionally, the expression patterns of POU2F3 and its related genes may further reveal the molecular characteristics of tuft cell-related cancer subtypes, offering theoretical support for precision medicine.

## Expression patterns of POU2F3 across different tumor types

**Figure 1:**
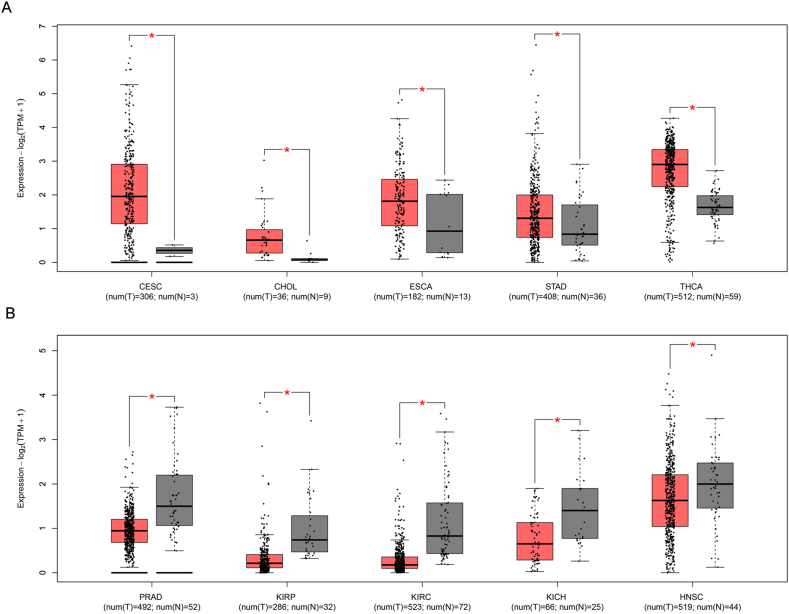
Differential POU2F3 expression in cancer versus adjacent normal tissues. **(A, B)** Box plots show POU2F3 expression differences between tumor and adjacent normal tissues, with up-regulation in cervical squamous cell carcinoma and endocervical adenocarcinoma (CESC), cholangiocarcinoma (CHOL), esophageal carcinoma (ESCA), stomach adenocarcinoma (STAD), and thyroid carcinoma (THCA), and down-regulation in prostate adenocarcinoma (PRAD), kidney renal papillary cell carcinoma (KIRP), kidney renal clear cell carcinoma (KIRC), kidney chromophobe (KICH), and head and neck squamous cell carcinoma (HNSC) (*P* < 0.05). Data were obtained and visualized using GEPIA (http://gepia.cancer-pku.cn).

**Figure 2:**
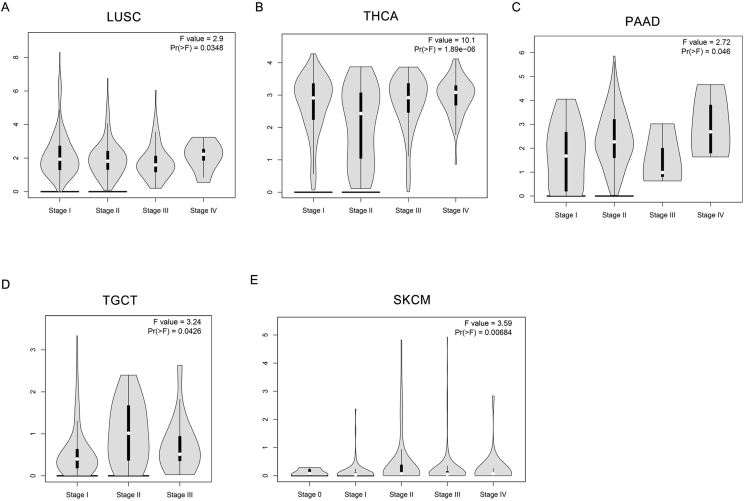
POU2F3 expression across different tumor stages. **(A)** In lung squamous cell carcinoma (LUSC), stage IV tumors exhibit significantly higher POU2F3 expression than other stages. **(B, C)** In thyroid carcinoma (THCA) and pancreatic adenocarcinoma (PAAD), stage IV tumors show the highest POU2F3 expression compared with earlier stages. **(D)** In testicular germ cell tumor (TGCT), POU2F3 expression is highest in stage II tumors, followed by stage III and stage I. **(E)** In skin cutaneous melanoma (SKCM), stage II tumors have the highest expression, while stage IV tumors show higher levels than other stages. Expression data were derived from TCGA via GEPIA (http://gepia.cancer-pku.cn).

This study analyzes the expression patterns of POU2F3 across 33 tumor types using TCGA pan-cancer data, providing a comprehensive overview of its expression across various cancers. Due to the lack of appropriate normal tissue samples, box plot analyses could not be performed for POU2F3 expression in mesothelioma and uveal melanoma. In contrast, analysis of the remaining 31 tumor types, using data from TCGA, revealed variable expression levels of POU2F3 in both tumor and normal tissues. Box plots were used to illustrate POU2F3 expression in each cancer type, including comparisons with matched adjacent normal tissues. The results showed that POU2F3 expression was significantly higher in tumor tissues compared with adjacent normal tissues, including cervical squamous cell carcinoma and endocervical adenocarcinoma (CESC), cholangiocarcinoma (CHOL), esophageal carcinoma (ESCA), stomach adenocarcinoma (STAD), and thyroid carcinoma (THCA) (Log_2_FC Cutoff: 0.2; *P* < 0.05; Fig. 1A). Notably, many of these cancers arise from luminal organs frequently exposed to microbial environments. This observation suggests that POU2F3 up-regulation in these tumors may be associated with interactions with the microbiota, potentially influencing tumor progression through immune modulation or epithelial differentiation pathways. In contrast, significantly lower POU2F3 expression was observed in tumor tissues relative to normal tissues in prostate adenocarcinoma (PRAD), kidney renal papillary cell carcinoma (KIRP), kidney renal clear cell carcinoma (KIRC), kidney chromophobe (KICH), and head and neck squamous cell carcinoma (HNSC) (Log_2_FC Cutoff: 0.2; *P* < 0.05; Fig. 1B). Further analysis of POU2F3 expression across different tumor stages revealed distinct, cancer-type-specific patterns. In lung squamous cell carcinoma (LUSC), stage IV tumors exhibited significantly higher expression compared with earlier stages (Fig. 2A). Similarly, in thyroid carcinoma (THCA) and pancreatic adenocarcinoma (PAAD) POU2F3 expression peaked in stage IV tumors (Fig. 2B and C). In testicular germ cell tumors (TGCT), the highest expression was observed in stage II, followed by stages III and I (Fig. 2D). For skin cutaneous melanoma (SKCM), stage II tumors exhibited the highest expression, with stage IV tumors also showing elevated levels compared with other stages (Fig. 2E). Collectively, these findings suggest that POU2F3 expression varies across tumor stages and may play a stage-specific functional role in cancer progression. However, with the exception of LUSC, THCA, PAAD, TGCT, and SKCM, most cancers that exhibit significant differences in POU2F3 expression between tumor and normal tissues do not display substantial variation across tumor stages. The up-regulation of POU2F3 in advanced-stage tumors may indicate that tuft cells adaptively promote tumor aggressiveness and immune modulation. Conversely, in other tumors, POU2F3 expression patterns may indicate involvement in early tumor development or potential suppression of malignant progression.

While POU2F3 alone was used to reduce inter-sample variability and avoid the ambiguity of multi-marker panels, we acknowledge the potential value of incorporating other tuft cell-associated genes. Thus, we examined the expression of five canonical tuft cell markers (GFI1B, AVIL, TRPM5, SOX9, and CHAT) alongside POU2F3 in ten representative cancer types. None of these additional markers exhibited consistent or significant differential expression between tumor and normal tissues (data not shown). By contrast, POU2F3 showed robust and reproducible differences, reinforcing its utility as a singular molecular classifier. Nevertheless, future identification of more specific tuft cell markers could improve the sensitivity and precision of tuft cell-like tumor classification, especially for clinical applications.

## Clinical features and prognosis of tuft cell-like tumors

**Figure 3:**
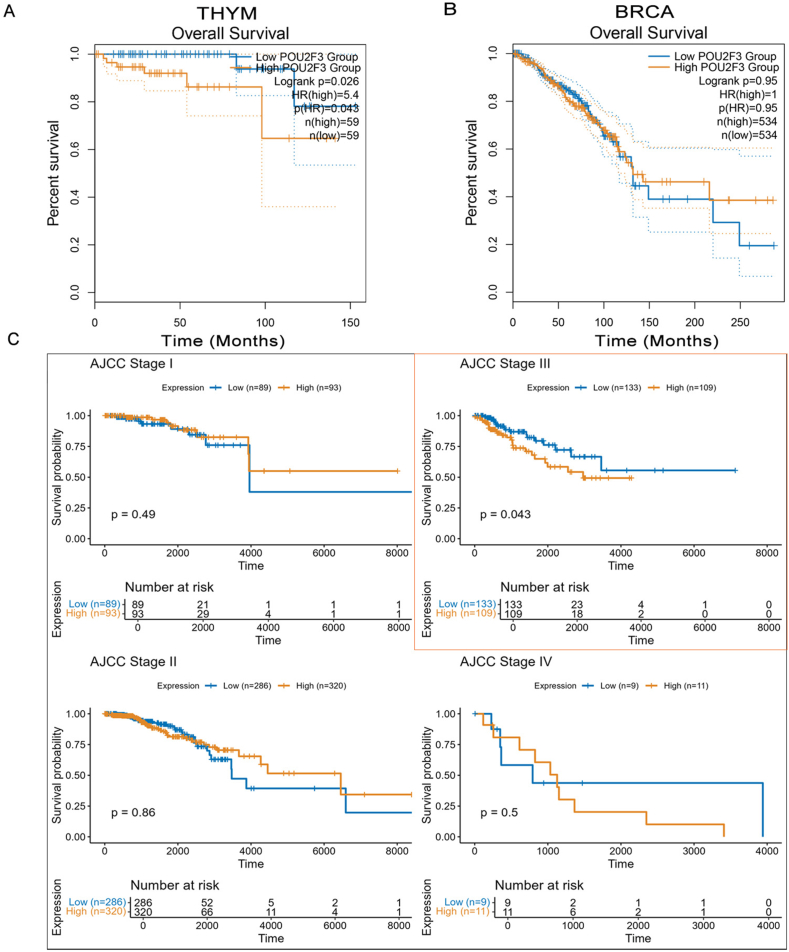
Clinical significance of POU2F3 expression in different tumors. **(A)** High POU2F3 expression is associated with shorter overall survival (OS) in thymoma (THYM). **(B)** No significant OS differences by POU2F3 expression were observed in the full cohorts of breast invasive carcinoma (BRCA). **(C)** Stratified survival analysis revealed that high POU2F3 expression could predict poorer OS in BRCA patients with stage III tumors.

To clarify the clinical relevance of tuft cell-like tumors, we compared survival outcomes between high and low POU2F3 expression groups using TCGA data. High POU2F3 expression was significantly associated with shorter overall survival only in thymoma (THYM) (Fig. 3A). No significant overall survival differences were observed in the overall cohorts of breast invasive carcinoma (BRCA) (Fig. 3B). However, stage-stratified Kaplan–Meier analyses revealed a stage-specific prognostic value: high POU2F3 expression predicted worse overall survival in BRCA patients with Stage III tumors (Fig. 3C). No such stage-specific associations were found in THYM. RNA-sequencing data were normalized as log_2_(count + 1), and POU2F3 expression was extracted using HGNC symbols. Patients were dichotomized into high and low expression groups based on the median. Clinical variables were standardized across cohorts, and survival analyses were performed using the Kaplan–Meier method and log-rank test. All analyses were conducted in R (v4.3.2) using survival, survminer, dplyr, and ggplot2. These findings suggest that POU2F3 expression varies across tumor stages, potentially reflecting a stage-specific functional role in tumor progression. The up-regulation of POU2F3 in advanced stages may indicate the adaptive response of tuft cells in promoting tumor aggressiveness or immune modulation as the tumor evolves. Conversely, in some tumors, POU2F3 expression patterns could imply early-stage tumor development or the suppression of malignant progression.

However, it is important to note that these analyses are based on TCGA RNA expression data, which offers several advantages, such as broad applicability across various tumor types and the ability to capture gene expression levels at a global scale. RNA expression analysis is a powerful tool for identifying potential biomarkers and exploring gene function in cancer. RNA levels may not directly correlate with protein expression or cellular activity, meaning that the observed POU2F3 expression patterns might not fully represent the biological functions of tuft cells in tumors. As RNA levels are not always reflective of functional activity, future research should incorporate proteomics and functional assays to provide a more accurate and comprehensive understanding of tuft cells' roles in cancer biology. This integrated approach will ensure a more robust exploration of POU2F3's impact on tumor progression and potentially identify new therapeutic targets.

## Mechanisms of tuft cell involvement in different tumors

### Small cell lung cancer and lung adenocarcinoma

In small cell lung cancer, a variant form that lacks neuroendocrine features has been found to express tuft cell markers, with POU2F3 identified as a key transcription factor regulating tuft cell identity in these tumors. This form of small cell lung cancer shows dependencies on POU2F3 and could serve as a potential therapeutic target.^18^^,^^19^ Moreover, POU2AF2 and POU2AF3 act as coactivators for POU2F3, regulating transcription at enhancer regions and maintaining tuft cell identity.^20^ Disruption of POU2AFs or targeting the SWI/SNF complex, crucial for chromatin remodeling, could affect gene expression and tumor growth, indicating that therapies targeting these pathways may be effective in tuft cell-like cancers.^21^ Additionally, tuft cell-like phenotypes are observed in thymic squamous cell carcinomas and some non-small cell lung cancer subtypes, including pulmonary squamous cell carcinoma and adenocarcinoma, which co-express POU2F3, GFI1B, and KIT.^22^ Although GFI1B is generally not expressed or is significantly down-regulated in most non-small cell lung cancer cases, these findings identify a distinct molecular subset that may inform personalized therapeutic strategies targeting tuft cell-associated markers. These findings highlight the potential of tuft cells to define new molecular subsets of lung cancers, thus informing the development of personalized therapies targeting these markers.

### Pancreatic adenocarcinoma

In pancreatic cancer, tuft cells form during acinar-to-ductal metaplasia in response to tissue injury. These cells have been observed in the pancreas under chronic inflammation conditions and in precursor lesions of pancreatic cancer. Research indicates a dual role of tuft cells in pancreatic tumorigenesis. On one hand, tuft cells have protective functions, such as producing prostaglandin D2 (PGD2), which inhibits the progression of pancreatic intraepithelial neoplasms.^23^ On the other hand, under the influence of molecular signals like Kristen rat sarcoma (KRAS) mutations and inflammatory cytokines such as IL-17A, tuft cells promote tumorigenesis.^24^ One article showed that tuft cells, absent in normal pancreas but present in precursor lesions, decrease as tumors progress. Using a dual recombinase lineage trace model, the researchers found that metaplastic tuft cells transdifferentiate into neural-like progenitor cells during carcinoma progression, which are linked to poor survival. Myc activity in tuft cells is crucial for this tuft-to-neuroendocrine transition, highlighting Myc as a potential therapeutic target for pancreatic cancer.^25^ Moreover, tuft cells, marked by the expression of DCLK1, contribute to tumor-associated stem cell-like features, including the ability to self-renew and resist therapy.^26^ The interaction between tuft cells and other cells in the pancreatic microenvironment, such as macrophages and the microbiome, complicates their role in tumor progression.^27^ While tuft cells may initially help maintain tissue homeostasis and suppress tumor formation, their aberrant activation can drive pancreatic cancer development. Understanding this complex role offers potential therapeutic targets for treating pancreatic cancer.

### Colorectal cancer

In colorectal cancer, tuft cells play a critical role in tumorigenesis and progression through their interactions with the immune microenvironment.^28^ Tuft cells promote immune escape in the tumor microenvironment by activating and releasing succinate, which stimulates epithelial cells and induces the infiltration of immune-suppressive cells like myeloid-derived suppressor cells. These cells secrete cytokines, such as IL-25 and IL-13, which further facilitate tumor progression.^28^ Additionally, tuft cells contribute to the formation of cancer stem cell-like features in colorectal cancer, particularly through IL17RB^+^ tuft cell-like cancer stem cells that express POU2F3 and promote tumor growth and metastasis.^29^ Targeting these cancer stem cells has been shown to significantly suppress tumor growth, suggesting that tuft cells may serve as a potential therapeutic target in colorectal cancer treatment.^29^ Additionally, tuft cells exhibit plasticity in response to intestinal injury or chronic inflammation. When the intestinal epithelium is damaged, some tuft cell precursors dedifferentiate and participate in regeneration and tumorigenesis, underscoring their pivotal role in colorectal cancer development.^30^

### Gastric cancer

Tuft cells play a significant role in the development and progression of gastric cancer, initially contributing to gastric metaplasia and dysplasia and exhibiting stage-dependent involvement throughout gastric tumorigenesis.^31^ Tuft cell populations increase in gastric conditions like Ménétrier's disease, Helicobacter pylori gastritis, and intestinal metaplasia. These conditions exhibit higher tuft cell numbers during the early stages of tumorigenesis, suggesting an association with precancerous lesions.^32^ A key mechanism in tuft cell involvement in gastric tumorigenesis is cholinergic signaling. Tuft cells and enteric nerves are major sources of acetylcholine (ACh) in the gastric mucosa, and the ACh–nerve growth factor (NGF) axis promotes NGF expression, which expands enteric nerves and promotes carcinogenesis. Inhibition of this pathway in models has reduced epithelial proliferation and tumor formation, suggesting it as a potential therapeutic target for gastric cancer prevention.^8^ Furthermore, tuft cells engage in crosstalk with group 2 innate lymphoid cells, supporting tumorigenesis. Tuft cell-derived IL-25 activates group 2 innate lymphoid cells, which release IL-13, leading to tuft cell hyperplasia. Disrupting this pathway can alleviate tumorigenesis in mouse models, offering a potential therapeutic strategy for early-stage gastric cancer.^33^

### Triple-negative breast cancer

In triple-negative breast cancer, tuft cells co-express SOX9, a key factor known to drive triple-negative breast cancer progression, suggesting a role in tumor aggressiveness.^34^ POU2F3-positive cells have been found in ductal carcinoma *in situ* and invasive breast cancer, indicating that tuft cells may be involved in early cancer development. Single-cell RNA sequencing has revealed that POU2F3-positive epithelial cells also express other tuft cell markers, such as SOX9 and AVIL, confirming the presence of tuft cells in the breast. The interaction between POU2F3 and SOX9 in tuft cell regulation warrants further investigation to better understand their role in breast tumorigenesis, especially in triple-negative breast cancer, and explore potential therapeutic targets for this aggressive cancer subtype.^34^

### Prostate cancer

The role of tuft cells in prostate cancer is still being explored, with initial evidence suggesting a complex involvement in tumor development. Tuft cells may contribute to tumorigenesis in response to tissue injury, chronic inflammation, and genetic mutations.^35^ Further research is needed to elucidate the precise mechanisms through which tuft cells influence prostate cancer progression and their potential as therapeutic targets.

### Neuroendocrine neoplasms

Tuft cells and neuroendocrine neoplasms represent two distinct, yet overlapping, cellular entities that have garnered increasing attention in cancer research. Recently, tuft cell-like tumors have been identified in various neuroendocrine malignancies, including pulmonary and extrapulmonary neuroendocrine carcinomas, which exhibit gene expression signatures resembling those of tuft cells.^36^^,^^37^ One study demonstrated that POU2F3 was expressed in a subset of neuroendocrine marker-low small cell lung cancer, where it serves as a diagnostic marker. Additionally, POU2F3 expression was observed in some cases of large cell neuroendocrine carcinomas and basaloid squamous cell carcinoma.^38^ Another study showed that tuft cell-like small cell lung cancer and pulmonary large cell neuroendocrine carcinoma exhibited co-expression of neuroendocrine markers (*e.g.*, NCAM1) and squamous markers (*e.g.*, KRT5), with additional features such as BCL2 and KIT expression.^39^ This hybrid nature has implications for tumor classification, prognosis, and therapy. A study has highlighted that tuft cell-like cancers often exhibit lineage ambiguity, with distinct clinicopathologic features such as high MYC expression and susceptibility to targeted therapies like PARP and BCL2 co-inhibition.^39^ These findings underscore the potential therapeutic vulnerabilities of tuft cell-like neuroendocrine tumors and suggest that further exploration of tuft cell biology in neuroendocrine neoplasms could provide novel insights into their pathogenesis and treatment strategies. Moreover, the recognition of tuft cell-like features in various cancer types may pave the way for new diagnostic and therapeutic approaches, particularly in tumors where traditional neuroendocrine markers are insufficient for precise classification.

Table 1 summarizes the roles of tuft cells in various tumors based on their tumor-promoting and tumor-suppressing mechanisms, integrating molecular pathways and key effectors. TCGA expression data for POU2F3 across different cancers were analyzed to highlight expression patterns, providing insights into tuft cell-associated tumorigenesis and potential therapeutic targets.

**Table 1:** Tuft cell roles, mechanisms, and expression patterns in various tumors.

Tumor type	Tumor-promoting role	Tumor-suppressing role	Key effectors	References
Small cell lung cancer & lung adenocarcinoma	POU2F3^+^ subtype of small cell lung cancer depends on tuft cell identity; POU2AFs and SWI/SNF complex maintain tuft cell phenotype; tuft cell-like markers co-expressed in non-small cell lung cancer subtypes.	Not well established	POU2F3, POU2AFs, SOX9, ASCL2, IGF1R, KIT, GFI1B	18, 19, 20, 21, 22
Pancreatic adenocarcinoma	KRAS mutations and IL-17A induce tuft cell-mediated tumorigenesis; tuft cells contribute to tumor stemness and therapy resistance.	PGD2 production inhibits pancreatic intraepithelial neoplasm progression; loss of tuft cells accelerates fibrosis and tumorigenesis.	Pou2f3, DCLK1, IL-17A, KRAS, PGD2	23, 24, 25, 26, 27
Breast invasive carcinoma	SOX9^+^ tuft cells drive tumor aggressiveness; tuft cells may contribute to early breast cancer development.	Not well established	POU2F3, SOX9, AVIL	34
Colorectal cancer	Succinate release induces myeloid-derived suppressor cell-mediated immune escape; IL-25/IL-13 signaling promotes tumor progression; tuft cell-derived cancer stem-like cells drive metastasis.	Some tuft cell precursors dedifferentiate for tissue regeneration.	POU2F3, IL-25, IL-13, myeloid-derived suppressor cells, IL17RB^+^ tuft cell-like CSCs	28, 29, 30
Gastric cancer	Cholinergic signaling (acetylcholine (ACh)-nerve growth factor (NGF) axis) promotes carcinogenesis; tuft cell hyperplasia supports tumorigenesis via IL-25/IL-13.	Tuft cells decline in poorly differentiated tumors, suggesting loss of tumor-suppressive function.	ACh-NGF axis, IL-25, IL-13, *H. pylori* gastritis, intestinal metaplasia	8,32,33
Prostate cancer	Potentially linked to chronic inflammation and genetic mutations in tumor initiation	Not well established	Under investigation	35
Neuroendocrine neoplasms	Hybrid tuft cell-neuroendocrine tumors exhibit high MYC expression and lineage ambiguity; tuft cell-like small cell lung cancer and pulmonary large cell neuroendocrine carcinoma show BCL2 and KIT expression.	Not well established	POU2F3, BCL2, KIT, NCAM1, MYC	36, 37, 38, 39

## Optimization of the tumor classification system associated with tuft cells

To refine the classification of tuft cell-associated tumors, future research should integrate advanced technologies such as single-cell RNA sequencing and spatial transcriptomics. These approaches will provide a more detailed characterization of tuft cell populations within the tumor microenvironment, revealing their functional heterogeneity and dynamic roles in tumor progression. By mapping gene expression at both the single-cell level and spatially distinct regions of tissues, researchers can uncover distinct tuft cell subpopulations and their interactions with other cellular components, offering a refined framework for tumor classification.

In addition to technological advancements, identifying novel tuft cell markers is crucial for improving the specificity and accuracy of tumor classification. While POU2F3 is the most widely recognized marker, additional markers need to be explored to distinguish tuft cells from other epithelial or stromal cell types across different tumor contexts. Developing optimized immunohistochemistry markers and molecular signatures will not only enhance tumor diagnosis but also facilitate the stratification of patients based on tuft cell-associated tumor characteristics, aiding in personalized treatment approaches.

In the long run, a comprehensive understanding of tuft cell-related biomarkers and their roles in tumorigenesis could lead to the development of novel diagnostic tools and targeted therapeutic strategies. This will ultimately improve the clinical management and outcomes of patients with cancers driven by tuft cell dysfunction or transformation.

## Discussion

This study highlights tuft cells as emerging diagnostic and therapeutic targets across diverse cancers. Tuft cells, initially characterized as chemosensory epithelial cells with roles in immune modulation, have recently been implicated in the pathogenesis of multiple malignancies. Tuft cells, defined by the transcriptional regulator POU2F3, represent a lineage-specific epithelial population whose role in tumorigenesis has only recently been appreciated. Emerging evidence implicates these cells in tumor initiation and maintenance in select epithelial cancers and highlights their potential as biomarkers and targets for precision therapy.^18^,^21^,^36^

This study underscores the complex role of tuft cells in the tumor microenvironment. While tuft cells are often associated with immune responses and epithelial differentiation, their role in cancer progression appears to be dual-faceted, with both tumor-suppressive and tumor-promoting effects. For instance, in pancreatic ductal adenocarcinoma, tuft cells initially act as a protective mechanism against tumor formation through the secretion of PGD2.^23^ However, under the influence of inflammatory cytokines or KRAS mutations, tuft cells may also promote tumorigenesis by transdifferentiating into neural-like progenitor cells, which are associated with poor survival.^24^ This dynamic suggests that tuft cells could have stage-specific roles in cancer, either supporting tumor progression or inhibiting it, depending on the molecular context.

The capacity of tuft cells to undergo transdifferentiation into more aggressive phenotypes, including neuroendocrine-like cells, in PDAC underscores their remarkable cellular plasticity and potential contribution to tumor progression. The role of Myc in this transition is particularly noteworthy, as it could serve as a critical therapeutic target.^25^ In malignancies such as gastric cancer, tuft cell-like phenotypes also contribute to immune modulation, promoting immune escape by activating myeloid-derived suppressor cells and secreting IL-25.^32^ Together, these findings highlight tuft cells as key regulators of both tumor plasticity and immune escape mechanisms.

POU2F3 is a master regulator of tuft cell identity and differentiation, making it a central marker for tuft cell-like tumors. This study shows that POU2F3 is crucial for tuft cell specification and differentiation, making it a valuable tool for identifying tuft cell-associated malignancies. Its expression patterns are notably up-regulated in cancers that originate from epithelial cells exposed to luminal environments, such as CESC and STAD (Fig. 1). These findings suggest that tuft cell-related tumorigenesis may, in some cases, be influenced by environmental factors such as microbiota. However, we acknowledge that for tumor types like bladder cancer, CHOL, PAAD, and THCA, direct evidence linking microbial exposure to POU2F3 regulation is currently lacking. Tuft cells are known not only for their chemosensory functions but also for their involvement in immune signaling, epithelial remodeling, and cellular plasticity.^5^^,^^7^ Therefore, the functional relevance of POU2F3 up-regulation in these cancers may reflect non-microbial roles of tuft cells, and future studies will be necessary to clarify the underlying mechanisms. However, the expression of POU2F3 across different tumor stages reveals stage-specific roles, with higher expression observed in advanced cancers, potentially reflecting an adaptive response to tumor progression. This variability emphasizes the need for further investigation into how POU2F3 expression impacts tumor aggressiveness and its potential as a prognostic marker.

The association between POU2F3 expression and clinical outcomes appears to be cancer- and stage-specific rather than universally consistent. In THYM, high POU2F3 expression was significantly associated with shorter overall survival, supporting its role as a marker of poor prognosis in this tumor type. By contrast, in BRCA, no survival differences were observed in the overall cohort; however, stage-stratified analyses revealed that high POU2F3 expression predicted worse outcomes specifically in patients with stage III tumors. These results suggest that the prognostic value of POU2F3 may emerge only at certain stages of tumor progression, reflecting context-dependent biological functions of tuft cell-like programs. The up-regulation of POU2F3 in advanced BRCA could imply an adaptive role in driving tumor aggressiveness or modulating the tumor microenvironment as the disease evolves. Collectively, these findings underscore the complexity of POU2F3 as a biomarker and highlight the necessity of tumor type- and stage-specific interpretation in clinical settings.

Accumulating evidence has shed light on the mechanistic underpinnings of tuft cell involvement in tumorigenesis, providing a comprehensive understanding of how tuft cells influence various cancer types. In small cell lung cancer, tuft cells co-express neuroendocrine markers and exhibit dependency on POU2F3 ^38^. This suggests that tuft cells may contribute to a subset of lung cancers with a hybrid phenotype, combining both neuroendocrine and epithelial traits. In PDAC, the dual role of tuft cells is evident, with their protective functions during early stages of tumorigenesis contrasting and with their tumor-promoting activities in later stages.^23^^,^^24^ The ability of tuft cells to adopt neural-like characteristics in response to oncogenic signals underscores the complex interactions between tuft cells and the tumor microenvironment.

It remains unclear whether tuft cell-like tumors truly originate from bona fide tuft cells or represent a de-differentiated epithelial state that mimics tuft cell features. Current evidence supports both possibilities. In colorectal cancer, DCLK1^+^ tuft cells have been shown to be long-lived and quiescent under homeostatic conditions, yet capable of initiating tumorigenesis following inflammatory injury, as demonstrated by lineage tracing.^40^ In PDAC, metaplastic tuft cells can transdifferentiate into neural-like progenitor cells, which are linked to poor prognosis.^25^ These findings suggest that tuft cells may act as a latent reservoir for malignant transformation in certain contexts. However, in many other tumor types expressing POU2F3, there is no direct evidence supporting a tuft cell origin. It is possible that the tuft cell-like phenotype observed reflects cellular plasticity or lineage reprogramming, rather than a true developmental lineage. Clarifying this distinction through integrative transcriptomic, epigenetic, and functional studies may provide key insights into tumor cell identity and offer novel therapeutic opportunities.

In colorectal cancer, tuft cells promote immune escape by activating myeloid-derived suppressor cells and secreting cytokines like IL-25 underscoring their role in shaping the tumor immune microenvironment.^28^, ^29^ These immunomodulatory functions raise an important question: Does POU2F3 expression in tuft cell-like tumors influence therapeutic responsiveness, particularly to immunotherapy? Although no direct evidence currently links POU2F3 to immunotherapy outcomes, emerging data from small cell lung cancer subtypes provide suggestive insights. Notably, the small cell lung cancer subtype lacking expression of ASCL1, NEUROD1, and POU2F3 is characterized by an inflamed gene signature and has shown a better response to immune checkpoint blockade.^41^ In contrast, POU2F3-positive small cell lung cancer exhibits lower immune infiltration and a poorer response to immunotherapy, implying a potential association between high POU2F3 expression and immune evasion. However, in a recent clinical study of extensive-stage small cell lung cancer, while Yes-associated protein 1 (YAP1) expression was negatively correlated with immunotherapy efficacy, POU2F3 showed no significant association.^42^ These findings suggest a context-dependent role of POU2F3 in modulating tumor-immune interactions. Further investigation is warranted to determine whether POU2F3 could serve as a predictive biomarker of immunotherapy resistance. Integrating immunogenomic data with spatial and functional profiling may help clarify this relationship across different tumor types.

Moreover, the identification of novel tuft cell markers beyond POU2F3 could improve the specificity and accuracy of tumor classification. As tuft cell-like tumors often exhibit molecular features overlapping with other tumor types, such as neuroendocrine neoplasms, a refined diagnostic approach is needed to distinguish tuft cell-like cancers from other malignancies with similar gene expression profiles.

While POU2F3 was used as the central marker for defining tuft cell-like tumors in this study, we recognize the limitation: the absence of other canonical tuft cell markers (*e.g.*, GFI1B, AVIL, TRPM5, SOX9, and CHAT) in our classification framework. In our TCGA-based analysis, these additional markers did not show consistent or reproducible expression differences across tumor types. Importantly, when incorporated alongside POU2F3, they did not improve—and in fact reduced—the ability to distinguish tumor from normal tissues. For this reason, we relied exclusively on POU2F3, which alone provided a more robust and reproducible classifier of tuft cell-like tumors. Nevertheless, this reliance on a single marker may oversimplify the molecular identity of tuft cell-associated tumors, and future studies will be required to validate additional markers and refine the classification system.

## Conclusion

This study advances our understanding of tuft cells and their role in cancer biology, particularly through the lens of POU2F3 expression. By systematically classifying tuft cell-like tumors and analyzing their clinical relevance, we can develop better diagnostic tools and therapeutic strategies for cancers driven by tuft cell dysfunction or transformation. Future research should focus on elucidating the molecular pathways governing tuft cell plasticity and exploring their potential as therapeutic targets in personalized cancer treatment. Ultimately, a comprehensive understanding of tuft cell biology will contribute to improving patient outcomes in cancers where tuft cells play a central role in tumor progression.

## CRediT authorship contribution statement

**Mengling Ye:** Writing – review & editing, Writing – original draft, Formal analysis, Conceptualization. **Yuyang Liu:** Formal analysis. **Hui Li:** Writing – review & editing, Funding acquisition, Conceptualization.

## Funding

This study is supported by the Guangxi Science and Technology Base and Talent Special Project (No. GUIKE AD22035047) and the University of Virginia Cancer Center Support Grant (No. P30 CA044579).

## Conflict of interests

Hui Li is the member of Genes & Diseases Editorial Board. To minimize bias, he was excluded from all editorial decision-making related to the acceptance of this article for publication. The remaining authors declare no conflict of interest.
